# Fathers’ experiences of care when their partners suffer from peripartum cardiomyopathy: a qualitative interview study

**DOI:** 10.1186/s12884-018-1968-x

**Published:** 2018-08-13

**Authors:** Harshida Patel, Marie Berg, Cecily Begley, Maria Schaufelberger

**Affiliations:** 1 Institute of Health and Care Sciences, Sahlgrenska Academy, University of Gothenburg, Gothenburg, Sweden; 2 Centre for Person-Centred Care (GPCC), University of Gothenburg, Gothenburg, Sweden; 3 School of Nursing and Midwifery, Trinity College Dublin, Dublin 2, Ireland; 4 Department of Molecular and Clinical Medicine, Institute of Medicine, Sahlgrenska Academy, University of Gothenburg, Gothenburg, Sweden

**Keywords:** Peripartum cardiomyopathy, Fathers´s experiences, Pregnancy, Heart failure, Qualitative study, Information, Care

## Abstract

**Background:**

Peripartum cardiomyopathy (PPCM), a potentially life-threatening condition in women, can have a profound impact on the family. Although structured support systems are developed, these systems tend to be based on the healthcare providers’ perceptions and focus mainly on mothers’ care. Fathers’ vital role in supporting their partners has been advocated in previous research. However, the impact of PPCM on the male partners of women is less understood. The aim of this study was to explore the experiences of healthcare in fathers whose partner was suffering from peripartum cardiomyopathy.

**Methods:**

The data from interviews with fourteen fathers were analysed using inductive content analysis.

**Results:**

An overarching category *“The professionals could have made a difference”* was identified from the data, characterised by the sub-categories: ‘*To be informed/not informed,’ ‘To feel secure/insecure,’ ‘To feel visible/invisible’* and *‘Wish that it had been different’.* Lack of timely information did not allow fathers to understand their partner´s distress, and plan for the future. The birth of the child was an exciting experience, but a feeling of helplessness was central, related to seeing their partner suffering. A desire for follow-up regarding the effect of PPCM on themselves was expressed.

**Conclusions:**

When men, as partners of women with PPCM, get adequate information of their partner´s condition, they gain a sense of security and control that gives them strength to handle their personal and emotional life-situation during the transition of becoming a father, along with taking care of an ill partner with PPCM. Hence, maternity professionals should also focus on fathers’ particular needs to help them fulfil their roles. Further research is urgently required in this area.

## Background

Peripartum cardiomyopathy (PPCM) is a life threatening condition and defined as*: An idiopathic cardiomyopathy presenting with heart failure (HF) secondary to left ventricle systolic dysfunction towards the end of pregnancy or in the months following delivery, where no other cause of HF is found. It is a diagnosis of exclusion. The left ventricle may not be dilated but the ejection fraction is nearly always reduced below 45%* [1]. The incidence is increasing in the western world [2–4] and in Sweden the prevalence is 1:5719 births [5]. PPCM is much more common in other countries, e.g., 1:1000 live births in South Africa and up to 1:300 live births in Haiti [6]. The diagnosis of PPCM in women can be a transition trigger for fathers, exacerbating the disruptions created in transition to fatherhood. Becoming a father is one of the biggest changes a man can experience in his life, and even normal transition to fatherhood has been shown to be a stressful time for men [7].

In Sweden, policy enables fathers to take paternal leave, with an allowance, instead of the mother [8]. Such policy practices clearly provide a supportive structure for fathers’ transition to a new role, and have been advocated in the international arena. The World Health Organization (WHO) [9] calls for a broader understanding from healthcare professionals of paternity and men's needs and perspectives related to perinatal care. A meta-synthesis, with data from different continents between 2000 – June 2013 with 120 fathers, showed that fathers wish to be included during labour and birth, to support their partner in an adequate manner [10]. Despite this, there appears to be a lack of awareness and recognition by health professionals of the benefits of effective paternal involvement in maternity care [11]. In fact, one study has reported that support systems for mothers tend to increase over time whereas, for fathers, support systems decrease [12]. The fathers are expected to be involved in pregnancy, childbirth and care of the new-born, but are also expected to hide their own feelings of anxiety, anger, sadness and fear, especially if those feelings might upset the mother with serious illness [13].

PPCM is associated with pregnancy and may be difficult to diagnose because of overlapping symptoms of HF with those of pregnancy. The women sometimes may fully recover to normal heart function, can live a normal life with medication, or progress to severe HF requiring heart transplantation [3, 4]. Heart failure is a recurrent risk in another pregnancy, even if the heart recovers [14].

Women´s potentially life-threatening complications during pregnancy or after a birth complicated by PPCM can have a profound impact on the whole family. Fathers, following their partner’s complicated childbirth have described their sadness and disappointment that pregnancy and childbirth has not been as expected [15–17]. Symptoms and experiences in PPCM mothers have also been described [18, 19]. Despite the near universal attendance of fathers at childbirth there is comparatively little research on fathers’ experiences; specifically, their experiences in the perinatal period complicated by their partner’s PPCM diagnosis has not been described. An understanding of the father’s experience is important to the professional, with the realisation that the father plays a crucial role for his children and also supports their partner.

### Aim

The aim of the study was to explore the experiences of healthcare in fathers whose partner was suffering from peripartum cardiomyopathy.

## Method

A qualitative research design was used, with data collected through interviews and analysed using inductive content analysis as described by Elo and Kyngäs. This method was selected as it has a greater focus on the unexplored phenomenon and allowed us to be open-minded, thus generating findings as close as possible to the data supplied, consistent with our constructivist standpoint [20].

### Data collection

During interviews with 19 women from western Sweden about their symptoms of PPCM and experiences of healthcare, reported earlier [18, 19], recruitment of the partners took place, using purposive sampling. The women were asked for permission to contact their partner, the child´s father. Two women had separated and had no contact with their partners and seventeen women agreed to provide their partner´s cellphone number through which the lead researcher (HP) contacted them. Of these, fourteen agreed to participate in the study. Three out of seventeen declined to participate, two because of lack of time and one did not want to be involved. Eight were first time fathers. The participating fathers received information about the purpose of the study both in oral and postal form. Some had queries, which were answered prior to signing the consent forms. The interviews were arranged at a mutually agreed, convenient time and place and carried out during 2012 – 2013; seven were done in person at the research clinic and seven by telephone. The semi-structured interviews were carried out using a guide, similar as the one used for women with PPCM, and some brief demographic data were also collected prior to interview. The open question was: “Will you tell me about your experiences of healthcare when your partner was suffering from PPCM?” The fathers were encouraged to speak freely. During the interviews, the interviewer (HP) asked questions for clarification, such as: “What do you mean?” or “Could you say something more about that?” The interviews lasted 20 – 73 minutes, were audiotaped and transcribed verbatim. Data accuracy was ensured by comparing transcripts with the audiotapes, and relevant information from field notes and memos, such as non-verbal cues or reactions, were noted [21]. Data were stored on a password-protected computer.

### Data analysis

The inductive content analysis [20] aimed at extracting and identifying central aspects about the fathers´ experiences of healthcare involved active reading, verifying, correcting, modifying and organising the data. Initially, HP and MB read all the transcripts to get an overall ‘feel’ for the data. In the next step, the text was reread and sections relating to the research question were identified in each interview transcript. Small sections of text explaining features relating to the study phenomenon were marked and summarising marginal notes were made. In the next step, these “units of analysis” including the notes were copied onto independent sheets, and headings summarising the descriptions of healthcare, i.e. codes, were added. Further grouping of codes was done, based on similarity, into higher order headings, i.e., categories. The categories were revised throughout the process by either deleting or collapsing under a new heading as the analysis progressed, until a consensus was reached. The final analysis identified four categories forming one main category. These findings were individually and then collectively discussed (HP, MB, CB). The final decisions were made through discussions and reconciliation of any differences and divergences among the authors. Reliability checks were made by moving back and forth between data and the formed categories. Participants were not asked for feedback on the analysis, to avoid work overload at a time they were very busy.

## Results

The age of the 14 participating fathers varied between 26 and 44 years old (M=34.5; SD 5.7). Four had high school, seven had college, and three had university education of more than 3 years. All were married or cohabiting; 12 were employed and two had their own business. Time ranged from 3 – 190 days from symptom start to PPCM diagnosis in their partner, and time from PPCM diagnosis to the interview ranged from 3 months to 7 years.

The analysis revealed a main category summarising the fathers’ experiences of healthcare: “*The professionals could have made a difference*”, which was characterised by four sub-categories described below, including illustrating excerpts. The example of identification of sub-categories and categories from the data is described in Table 1.

**Table 1:** Examples of categorisation process

Unit of analysis	Codes	Sub-category	Main category
You did not get the explanation without asking; what? How? You just need to sit down with a doctor…to go through the disease, causes and treatment plans.	Desire a clear and complete picture of the situation	To be informed/not informed	The professionals could have made a difference
You are desperately searching for each straw that can testify the improvement (in woman´s condition) and when you hear that "you may consider yourself chronically ill because now it has gone so far and you have not shown any improvement"	The way of communication is crucial		
Will she die? Be disabled? Will she survive? You need a quick conversation with the doctor to avoid frustration	Several unpleasant thoughts appear in your mind and you need quick information		
I think they could have facilitated so I would have been more prepared to help my partner	Understanding the situation facilitates security	To feel secure/ insecure	
The doctors said it looks good but a nurse said the women rarely get well from this	Incorrect information creates insecurity		
The main problem was the lack of collaboration between different specialties that led to the missing or erroneous drug administration.	Coordination between specialties is necessary to secure patient safety		
It almost felt that it was a hassle for the staff at the ward that she was there and I was there too with her	Not feeling welcome created feelings of insecurity		
She was very serious and was close to death… It felt safe because she was surrounded by a good team	A whole team of people around her conveyed security		
They (neonatal ward) did not ask me as father about anything, then you really had to try to drag their attention.	Had to make great efforts to become a part of own babies’ care	To feel visible/invisible	
You accept yourself like a spectator. Then you realize that, ´I'm also a human, not robot, I also have my needs. Talk to me too. I also have own feelings´.	Desire to be visible		
At the ward it was strenuous. I had to go out to get my own food even though I was there to take care of the child	Invisible carer		
Perhaps they (healthcare professionals) have had targeted action against the couple together. She felt I was not there for her, but I think I supported her. This leaves their marks as well.	Experience of being invisible from the partner		
There was both fear but also joy over the new born baby. Everything was up and down, It was very difficult situation.	We had looked forward to having joyous arrival of the baby but it was not as expected	Wish that it had been different	
Did not have time to think about your own health. Taking care of two children, work, shopping, school/ kindergarten and making food.	Don´t have time to think about own health. Trying to balance between different priorities		
You feel rejected and it was difficult. They didn´t pay attention to whatever you say (about woman not feeling well). In the end you give up. There was no point to nag.	Wish adequate encounter with healthcare professional		

### To be informed/not informed

‘To be informed/not informed’ was a sub-category related to how much control fathers had over the situation. Support was needed by being given information by professionals. Some satisfaction over the healthcare received was expressed; however, information provided was not sufficient. Fathers argued that communication was one of the ways to gain knowledge and understand the meaning of HF, the future, and forecast how best to support their partner. Good information for the fathers involved was, for example, a good targeted conversation with the doctor along with the rest of the family, about the prognosis and consequences of HF. One father told about his best experience as follows: "*The doctor invited our parents to inform them about my wife’s condition"* (P2).

Fathers across the study felt that healthcare professionals were honest, open and frank about the details of PPCM diagnosis, the woman´s health status, prognosis, consequences and the future strategy. Fathers described how they were left alone, when professionals were busy caring for the mother; this was a stressful and anxiety-provoking moment as they felt cut off from the information they needed about their wife´s condition. Fathers with negative experiences often became distressed recalling conversations with healthcare professionals. One of the fathers said:

*“We did not get the explicit information in spite of asking the same question several times…thoughts can go in all directions. Just needed to talk to someone, who could answer…they (professionals) could have made it easier” (P5).*

A wish to participate in the woman´s care was expressed; in order to be responsive to the woman´s need, but to do this they required adequate information. Some fathers perceived professionals to be ‘too busy’ to take the time to inform and explain. One father´s statement echoed others’ views:

“*The staff informed me after the things were done, but if I would have known about it earlier, I could have been more prepared…there are skilled nurses, but with limited power to convey the medical information”* (P10).

Some others said, “*I still have many unanswered questions*” (P4, P5, P10, P12, P20). //*“The unanswered questions are still spinning in my head. I want to know about relapse, if she can get affected by HF again without pregnancy?”*(P19).

A difficulty in putting all “the pieces” together was shown in the fathers´ descriptions. The memory of what happened was vivid:

“*I still can´t understand how they (post-natal ward) can discharge her with ongoing symptoms of shortness of breath, she could hardly take 2 – 3 steps and was swollen? No one reacted and took her seriously… the same night after discharge; we had to call several places because of deterioration, before she came to the right place…how come? Finally she was diagnosed with HF. I thought it was pneumonia and she will be fine in a few days. Heart did not exist in my wildest fantasy*” (P19).

Midwives’ competence and documentation routines were questioned by the fathers. Fathers were annoyed, as they felt a need to monitor every step taken in the caring of women. The knowledge gap in midwives was interpreted as frivolity (carelessness), because the unsatisfied fathers wanted to talk with the doctor for an explanation but the midwives did not understand the seriousness of women´s condition and, hence, did not refer fathers to visit the doctors.

Dissatisfaction was also noted due to the lack of collaboration between units in the hospital caring for women with serious conditions that resulted in a lack of important information about the woman´s condition being transferred. One of the fathers argued that:

“*It seems that there are two different perspectives. Upon the arrival at ICCU ward, she was not allowed to put herself under even minimal strain, while just minutes before being sent from emergency…she was out in minus degrees with the baby and feeling herself dizzy, short of breath and exhausted. She already had a reduced heart capacity and needed to walk 500m…Until you have the right diagnosis, no one believes or takes you seriously*”(P11).

Although the fathers complained about insufficient, inexplicit and unintelligible information, they seemed to be happy about the woman´s recovery and follow-ups.

### To feel secure/insecure

The fathers´ feelings of security were partly associated with receiving thorough information, given in the language that they could understand, about everything that was happening around their partner; this helped them to avoid feelings of panic. Fathers’ caring attitude and empathetic presence during their partner´s illness period was a huge resource for both the mother and new-born as well as elder siblings at home. A good encounter with healthcare professionals was experienced positively, although with some negative points.

“*A lot of people around provided security but no opportunity for the relationship. You want to be involved in healthcare …but then you need someone that you feel secure with to ask…*” (P18).

Feeling secure appeared to be associated with gaining deeper knowledge about the reasons for PPCM in order to understand the woman, and eased the coping process. The fathers did not want to be spared from the details about their partner’s condition regardless of reason, e.g. “*no one has told us about how close she had been to death*” (P6), // “*there was a risk, for she was close to dying*” (P7), // “*I thought more or less she would die" (*P10). The fathers expected to receive realistic information. For example, they hoped for heart recovery, but also wanted to hear about other undesired consequences such as, that she may suffer from depression. Another unsatisfied father said: "*Wish cardiologist had taken the time to inform me of what HF ​​is, about the future, risk of relapse and treatment”* (P4). On the contrary, there were also fathers who defended the professionals by saying: "*I understand that they could not give straight answers about the diagnosis…related to too little research of this condition (PPCM). They don´t know much about this either"* (P1, P12, P18).

Not knowing the meaning of HF led to a feeling of inadequacy and of being on the edge of the action without making needed input to support their partner in the best way. In addition, lack of awareness of the outcome of PPCM, uncertain future along with managing all the household responsibilities created feelings of frustration. One of the fathers mirrored others’ expressions:

“*You have to be superman, have strength to cope, argue and master the knowledge. You have to be healthy to be sick… wanted to get involved but there was no responsible doctor to talk with about wife´s condition…at the end you give up because…it was useless to keep up with nagging...”(*P9).

Sometimes fragmented information, or not really understanding the information given, seemed to be more confusing. For example, one father, whose partner had experienced recurrent pulmonary oedema and renal insufficiency, said:

“*There were endless unpleasant surprises…doctor explained what had happened, which led to further confusion about what led to what? What was wrong? We have no idea what happened? Why? Could it have been avoided? There are still unanswered questions probably as long as we live…still looking for the link between childbirth and HF, but have not been able to solve the puzzle yet…I wish I could have had more support” *(P12).

Another father, confronted with the words “heart transplantation,” was emotionally destroyed. In spite of contradictory feelings (related to joyous news of twin births and the sad news of wife´s PPCM) he explained:

“*When you lack knowledge, it´s better to leave it to the experts…the surprises never end. First, mental preparation for heart transplantation and prolonged hospitalisation but suddenly the next day, you hear that she is going home. This transition was difficult”* (P12).

The feelings of humiliation escalated in the father when his partner´s symptoms were explained as, "*you have not given birth before*" (P12). Sometimes, the message mediated differently by different professionals recalled bitter memories in fathers, and advised that professionals should be cautious in what they said to avoid making contradictory statements and causing anxiety for parents. For example:

*“When we were at the cardiac care unit… doctors came and said ‘this looks good and has a good prognosis.’ After a while one nurse came in and said, ‘there are rarely people get free from this (HF).´ We were sad to hear this as this was not the information we had received earlier…and now she (wife) is free from HF.”* (P3).

The uncertainty was caused by threat and desperation due to needing more information when they heard about PPCM. They immediately searched for more information on the internet and found some very worrying facts about HF. One of the statements reflected many others:

*“If you google ´heart failure´ it says only about the heart failure that older people have, basically, and they can die from it…and 50% chance of dying within one year and 90% chance in 3 years. Therefore, I was not happy…it was shocking and very strange that no one explained to me that pregnancy heart failure is different. There is no information on the Internet. I understood with time, but it took a long time to understand what HF actually is in women”* (P19).

Furthermore, the feelings of being ‘on thin ice’ and vulnerable were related to professionals’ ignorance, and their apparent lack of caring and interest in supporting the fathers, which led to feelings of insecurity. Their need for professionals to react sooner and more quickly in abnormal situations was also stressed. One frustrated father expressed his insecurity:

"*When we came again, they said, ‘oh well, what is it now? Are you back’? There was no warmth, comfort or security conveyed in the encounter*. *The doctor who came to check did not even reflect on her weight (>100 kg). He argued that it will disappear after discharge, but it did not. We had to seek emergency care again. Professionals at the post-natal care ward were confused, perplexed and had no idea how to act in this situation…luckily we were sent to the ICCU. The doctor examined her and gave her a diuretic, she began to pee...When they see that she could not urinate at postnatal ... doctor did not even take off the quilt and examine her... but said all the time that the problem will resolve"* (P6).

Moreover, the lack of space to accommodate fathers in health facilities was one of the issues experienced as unwelcoming and uncomforting. Fathers stressed understanding in this particular situation, "*you have a newborn child and the child's mother has been sick with PPCM...* "(P1). On the contrary one father, who had received this help found some relief: “… *a little comfort was found in the chaotic situation, when we received a private room in the neonatal unit*” (P18).

In spite of frustration and negative thoughts, the trust and faith in the professionals was obvious, once the woman had a diagnosis, which was described as:

*“My wife was in the ICU after re-operation because of bleeding…uterus was removed…complications…they (professionals) said renal failure and tachyarrhythmia; but I felt nonetheless the tranquility of knowing that they have everything under control”* (P12).

### To feel visible/invisible

Two different dimensions appeared in the majority of the interviews indicating fathers´ feeling of invisibility; from the health professionals and from the woman. The words like "sidelined", "ignored", "invisible" and "pushed aside" were used to describe feelings of invisibility.

To be visible meant that the staff were accommodating of the father’s presence. For example, when the professionals took time to explain the situation, were supportive, and gave a bed and food to the fathers. The ideals of involving both mother and father seemed to be important, and fathers’ inputs were regarded positively by professionals.

A feeling of invisibility was related to being uninformed and ill-equipped to understand what was going on with the woman. The disappointment that was present because their role of supporter and protector was endangered led to experiencing the feeling of being a spectator. The feelings of exclusion were evident when they heard about PPCM diagnosis from the woman herself, rather than by being told by health professionals. The lack of explanation from the doctors and midwives was experienced as “*helplessness, I couldn´t do anything*” (P20). To have to prepare and bring in their own food from outside the hospital, while taking care of the baby as well, was perceived as one of the aspects of “invisible fathers” (P1, P4, P18).

In general, fathers felt ‘visible’ when their presence was appreciated, but some of the fathers felt themselves to be in the way and pushed aside at the ward, perceiving that they were considered to be a "burden and hurdle"(P1, P2). One of the fathers stressed:

“*...even they (health professionals) should have seen that I am also a human being with my own feelings and needs, not a robot…talk to me, too! It was just my wife (who was in the focus) I also wanted to be seen. I felt bad, but could not tell that to my wife. I could not drag her further down with my worries too”* (P1).

“*I wanted answers on what was happening or a question posed to me about how I was doing. No one did that, not once. They are encouraging fathers’ presence during the whole process, but at the same time exhibit avoidance treatment”* (P2).

Being invisible also meant a sense of alienation. For example, one of the women was put on the transplant list without informing the couple. At the same time an ambiguous father mentioned: "*I wanted to be informed about everything, but I don´t know…had no time for all the information, due to taking care of twins*" (P12).

One of the fathers, whose baby was born disabled, was sad and expressed his thoughts related to the lack of support, and importance of being noticed by professionals:

“*Positive experiences from the healthcare team plays a major role in coping with the crisis…it took a long time to get to the normal level as we felt so low after childbirth and a deadly diagnosis of heart failure…I have not slept or had enough rest for a long time”* (P9).

The fathers developed increased stress thresholds, to handle the situation around childbirth and their partner´s illness, but also expressed feelings of loneliness: "*I have been sick ... no one talked to me afterwards*" (P6); and //“…*I wanted someone to think about me*” (P1, P5).

Another dimension of invisibility, in relation to their partner, included being “pushed aside,” which was “to be excluded by the woman” in their sexual relationship. For example, one of the fathers described: “*it happens often that she is too tired or does not want to kiss because of uneasy feelings of breathlessness*…” (P1).

Another aspect of ‘being recognised by professionals’ depended on the woman´s wish to involve the father, or not. Fathers felt sidelined, with feelings of alienation, belittlement and depersonalisation, as stated below:

“*I wanted to be informed, it was about me too. I did not find out when she was supposed to talk with the doctors, so I had no chance…no one noticed my presence*"(P4). // "*They did not ask me as a father about anything, you have to really work to drag their (professionals´) attention*” (P5).

### Wish that it had been different

Fathers expressed feelings of happiness over the birth of their child, but also mentioned that: “*wish that it had been different*”. The strongest memory was, “They did not take my wife seriously…she was questioned about her symptoms*”*. Having to watch their partner in pain was regarded as one of the most difficult things; to be present and not being able to help. The negative criticism was directed to the competence and attitude, expressed in the following quote:

*"I do not know if the doctors had any ideas either...If they would have had a better eye on her symptoms…maybe she shouldn´t have developed PPCM* (P3, P20). // “*I felt annoyance, sadness and the guilt of not being able to help her in a timely and sufficient way; related to their (professionals’) ignorance and for not questioning more. I was of course entirely in their hands. Think! if I'd the knowledge I would have argued with the doctors”* (P5)*.*

Fathers who had already been through childbirth before had noticed a change to poorer healthcare in the specialist childbirth unit (due perhaps to organisational change). First-time fathers had no idea, how it should be or could be. They described their feelings in similar words:

"*Until the diagnosis was made everything was very strange… I was angry…they (midwives) were not listening…she (wife) said several times, and I also expressed that she had difficulty breathing and could not pee…no reflection…We trusted them when they said ‘it will be all right’ ... It [anger] bubbles up in me even today for not resolving the issue at once*" (P6, P18, P19).

At time for the interviews all the fathers were actively working, and had altered their work schedule to some extent following the birth to enable them to take care of the family after the women´s PPCM was diagnosed. However, it was a challenge for some, particularly those who struggled to balance not only generating income but also taking care of the family, especially while their partner was sick for several months’ duration initially. For some fathers, the woman´s PPCM diagnosis led to concerns regarding their future career progression, and for others it led to compromise as they tried to reconcile and cope with the demands of a job and the need to be a care-taker. Some fathers were fortunate to receive family support while others, who had to adapt to the situation on their own, experienced it as lonely and unexpectedly burdensome.

### The professionals could have made a difference

The subcategories, ‘to be informed/not informed,’ ‘feeling secure/insecure’, ‘feeling visible/invisible’ and ‘wish that it had been different’ resulted in the main category, ‘the professionals could have made a difference.’ The findings revealed the miscellaneous experiences of healthcare described by the fathers. The fathers expected empathy and sensitivity from professionals but the majority felt “left out”. A trusting relationship was built when they were taken seriously, and their partner´s needs were met, which provided hope and feelings of security. Professionals who were reassuring, and took some time to talk with them, were appreciated. The professionals were found to be proficient but sometimes the situation was made more stressful than it really was because of lack of knowledge in the professionals and inadequate communication between involved partners from different units. The provision of information was found to be the central part and professionals in this context played a major role; but did not always succeed in mediating security or serve its purpose to calm the fathers because it was either not understood or needed repetition. The satisfaction about received information varied between specialties and professionals. For example, meeting with a cardiologist was appreciated because of the expertise; while midwives / gynecologists / obstetricians appeared to have limited knowledge about PPCM. Fathers received relatively little attention from the professionals as their suffering was often hidden from view in the wider picture of the woman´s needs due to PPCM. The fathers also needed confirmation and reassurance from professionals, to strengthen their feeling of being needed as a resource. Although they eventually accepted the situation, they wanted constructive help to work with their disappointment, anxiety and changed relationship.

## Discussion

This study aimed to explore the experiences of fathers whose partner was affected with PPCM. The findings highlight fathers’ experiences of healthcare related to the information, security and visibility. Four sub-categories; ‘To be informed/not informed,’ ‘Feeling secure/insecure,’ ‘Feeling visible/invisible’ and ‘Wish that it had been different’ were identified and resulted in the main category, ‘The professionals could have made a difference’ from patterns across coding sub-categories.

This is the first qualitative study about men’s experiences of healthcare while their partner is suffering from PPCM. Consequently, there are difficulties comparing and discussing the findings of the current study with previous research in the same context. Hence, the findings of the current study are discussed using transition theory [22, 23] from similar situations.

Results showed that being prepared and receiving clear information were essential elements of a positive experience that would support fathers to help their partner in the best way. The negative experiences were related mainly to feelings of being at the periphery due to lack of attention paid by professionals. The hardest thing for fathers to bear was the pain experienced by their partner and being unable to help. In this process, the professional´s encounter impacts on fathers’ ability to be emotionally and physically supportive to their partner. Similar findings have been described in studies of expectant fathers’ experiences of their partner’s labour and birth of their baby [10], men’s transition to fatherhood [24], and in postnatal care [25].

Transition to fatherhood is an emotionally challenging time; especially when their partner is ill and roles within the family are changing. A joint report of The Swedish Association of Obstetricians and Gynecologists and The Swedish Association of Midwives states the importance of fathers’ involvement in pregnancy but do not specify how a partner can be involved [26]. Nor does a report from the National Board of Health and Welfare [27] give any direction, but advocates the need for increased knowledge and understanding in professionals, about men's needs and perspectives in relation to pregnancy, childbirth, the care of the mother, and fatherhood [28].

Transitions are complex and multidimensional, a process that consists of moving from normal life, through an interim phase of psychological adaptation and reformation, to an assimilation of the new state of affairs into one’s inner and outer world [23, 24]. Previous studies have also shown that expectant fathers can find childbirth a traumatic time, during which they feel vulnerable, fearful for their partner and infant, and are in a transitional period that leaves them powerless, conflicted, and in limbo as a spectator [29, 30]. The research regarding the need to support fathers before, during and after birth appears to have become reflected in practice [31, 32]; however, the needs of fathers in situations like partner’s illness with PPCM, when the challenges may be the most demanding, are not always met. While each situation is unique, communication and genuine empathy are probably the most important keys to effective care in such situations.

In the current study, some fathers experienced a gradual role transition because of the length of time between symptoms starting and the PPCM diagnosis. The fathers’ dissatisfaction was associated with inadequate healthcare received by their partner before the PPCM diagnosis was made. Because the fathers did not directly understand the situation, frustrations arose related to uncertainty and insecurity. Johansson, in describing fathers’ experiences in intrapartum care, found that acting professionally facilitated feelings of security and control in the fathers [33]. In addition to the care that professionals provide for women, they also have a unique role and relationship with family members [34]. Giving information and making partners feel ‘visible’ are crucial factors to convey feelings of security and prepare fathers well for their expected role as a carer for their partner and the rest of the family. There was only one father who could report that their family members were invited to hear the information, demonstrating lack of family-centeredness in such a situation.

Meleis proposes that the ‘nature of the transition’ can facilitate or hinder the person’s ‘pattern of response’ [22].This is supported by the results of this study. Transition for fathers in our study began with enchantment and excitement in childbirth but was also associated with fear and grieving because of the woman’s symptoms related to PPCM illness and concern over the family’s future. Fathers re-evaluated their role as a supporter and carer in the family facing a devastating diagnosis like PPCM in their partner, and simultaneously standing up for the rest of the family, including caring for the new-born baby/twins. The weight of these transitions all occurring in a short space of time requires professionals’ deep understanding and thoughtfulness. It is of utmost importance to avoid father´s experience of feeling left out, by providing a real sense of being seen and heard by professionals, and by facilitating increased connectedness to the family.

The quality of the transition experience may be influenced by expectations, level of knowledge, environment, level of planning, and emotional and physical well-being [35]. Some of the fathers in our study experienced loneliness and suffered with women and wished the situation to be handled differently, whereas others were satisfied with the received healthcare. Being prepared for the transition was affected by fathers’ experiences of contradictory or frightening information and unpreparedness. Fathers in our study repeatedly stressed the importance of adequate information to avoid panic and ease the total situation. Journeying smoothly through a significant transition is facilitated by knowledge and preparation [23]. PPCM is one of the unexpected situations that might occur during this period, hence attentive and knowledgeable professionals are one of the essential requirements.

The fathers are not patient nor visitor, and are in between somewhere, acting as a carer and protector for the woman and their children. Professionals, by being attentive to fathers’ need may increase fathers’ self-confidence and help in developing strategies to handle the actual transition. Review research from fathers’ experiences during pregnancy and childbirth found that fathers experienced mixed feelings and required support from professionals and wanted to be involved and respected [36, 37]. Shorey [38] in a recent study described fathers’ postnatal experiences of an ‘emotional roller coaster’, being sleep-deprived, confused and not confident in taking care of their newborns and supporting their wives. The midwife's role is to provide support in the transition to parenthood and confusion can be avoided by increased awareness of fathers’ needs and by choosing information content and appropriate timing to convey in case of complications.

Ellberg et al. [39] studied fathers in postpartum care and showed that the father was treated as an outsider and care was described as ‘a woman's world.’ Hence, in order to be supportive, maternity professionals need to reflect on their work and shift focus towards family instead of only the woman. A great deal of the discontent with healthcare may be due to organisational failure, e.g. staff shortage, lack of knowledge about rare diagnoses like PPCM and lack of time. However, there is a need to develop strategies in how best to meet the father’s needs associated with when the woman is diagnosed with PPCM.

Although the need was apparent, some of the fathers expressed their ambiguity about seeking help. This attitudinal barrier may depend on the need for self-reliance in managing one’s own problems or a sense of resignation that nothing will help. These findings are in line with recent study findings among Australian fathers’ help-seeking behaviour [40]. Darwin et al [41] studied fathers’ views and experiences of their own mental health during pregnancy and the first ,postnatal year, and found that fathers may be reluctant to express their support needs or seek help amid concerns that to do so would detract from their partner's needs. Fathers in our study prioritised taking care of their baby and partner before their own needs of coping with the situation.

Fathers’ experiences in our current study can be compared to mothers’ experiences in our previous study from the same context [19]. Findings were similar regarding reports of satisfaction with healthcare but deficiencies in the encounter were found. The responses demonstrated that fathers’ expectations were closely related to those of their partners in line with previous research [19, 28, 42]. However, future research needs to explore this phenomenon in other related illnesses and cultural contexts.

Lastly, it is helpful for the woman´s wellbeing that her partner is acknowledged and valued as the father and partner, and not simply treated as a bystander. Previous studies describe how, when the staff ignored the father, both parents took offence [39]. It seems that fathers´ needs are similar regardless of maternity care context. The question arises as to how the situation is still the same, given that fathers’ needs for support have been identified in the literature for a long time.

### Strengths and limitations

A main strength of the current study is that this is the first study exploring fathers´ experiences of healthcare, when their partners are diagnosed with PPCM. Furthermore, the knowledge generated from this study fills a gap from a multidisciplinary viewpoint for better understanding and caring women with PPCM and their partners. Purposive sampling facilitated exploration of fathers’ experiences from within the same culture and context described in our earlier study on the experiences of women [18, 19]. Although data were collected from fathers specific to Sweden, the study contributes to the growing body of literature related to fathers’ experiences of healthcare in PPCM, given that this is a rare disorder. The retrospective nature of experiences generates issues of recall bias. Some fathers might not remember the whole picture as it was, because while looking back one can add, select or erase the meanings of earlier experiences. It is difficult to discuss this in the light of previous research because of limited knowledge exploration in this area. In general, the majority of fathers described similar experiences of healthcare, confirming data saturation. More studies are needed to understand tailored needs of fathers during such a transition from different cultures.

## Conclusion

This study provides empirical evidence on the father´s experiences and support needs during their partner´s PPCM. When men are supported, they gain a sense of security and control that gives strength to handle their life-situation of personal and emotional challenges during the transition of becoming a father along with taking care of an ill partner. Although midwives assess and ensure each mother´s successful transition, the need is acute to pay attention to the father´s transition in stressful situations where the joy is diminished by sorrowful news. It seems important for professionals to involve fathers and also the rest of the family in information after PPCM diagnosis, follow up interpretations of the information content, discuss expectations, and assess their experiences continuously during the woman´s recovery process. It is time now to ‘look outside the box’ to acknowledge the father´s need for well-being and reinforce his efforts because mothers rely on their partner for strength and support in such a critical condition. It is also important for professionals to be more effective care-givers from the gender equality perspective, and to notice both parents. Additional qualitative research with specific ethnic groups could further validate our findings and will enable targeted holistic and structured support through development of guidelines.

## Abbreviations

PPCM: Peripartum Cardiomyopathy
HF: Heart Failure
ICCU: Intensive Coronary Care Unit
ICU: Intensive Care Unit

## References

[1] Sliwa K, Hilfiker-Kleiner D, Petrie MC, Mebazaa A, Pieske B, Buchmann E, Regitz-Zagrosek V, Schaufelberger M, Tavazzi L, van Veldhuisen DJ (2010). Current state of knowledge on aetiology, diagnosis, management, and therapy of peripartum cardiomyopathy: a position statement from the Heart Failure Association of the European Society of Cardiology Working Group on peripartum cardiomyopathy. European Journal of Heart Failure.

[2] Kolte D, Khera S, Aronow WS, Palaniswamy C, Mujib M, Ahn C, Jain D, Gass A, Ahmed A, Panza JA (2014). Temporal trends in incidence and outcomes of peripartum cardiomyopathy in the United States: a nationwide population-based study. Journal of the American Heart Association.

[3] Pearson GD, Veille JC, Rahimtoola S, Hsia J, Oakley CM, Hosenpud JD, Ansari A, Baughman KL (2000). Peripartum cardiomyopathy: National Heart, Lung, and Blood Institute and Office of Rare Diseases (National Institutes of Health) workshop recommendations and review. Jama.

[4] Sliwa K, Hilfiker-Kleiner D, Petrie MC, Mebazaa A, Pieske B, Buchmann E, Regitz-Zagrosek V, Schaufelberger M, Tavazzi L, van Veldhuisen DJ (2010). Current state of knowledge on aetiology, diagnosis, management, and therapy of peripartum cardiomyopathy: a position statement from the Heart Failure Association of the European Society of Cardiology Working Group on peripartum cardiomyopathy. European journal of heart failure.

[5] Barasa A, Rosengren A, Sandstrom TZ, Ladfors L, Schaufelberger M. Heart Failure in Late Pregnancy and Postpartum: Incidence and Long-Term Mortality in Sweden From 1997 to 2010. *Journal of cardiac failure*. 2017; 10.1016/j.cardfail.2016.12.011 28069474

[6] Fett JD, Christie LG, Carraway RD, Murphy JG (2005). Five-year prospective study of the incidence and prognosis of peripartum cardiomyopathy at a single institution. Mayo Clinic proceedings.

[7] Chin R, Hall P, Daiches A (2011). Fathers' experiences of their transition to fatherhood: a metasynthesis. Journal of Reproductive and Infant Psychology.

[8] Ten days upon birth of a child [https://www.forsakringskassan.se/privatpers/foralder/vantar_barn/10-dagar/ ]

[9] World Health Organization: Working with individuals, families and communities to improve maternal and new born health; 2009.

[10] Johansson M, Fenwick J, Premberg Å (2015). A meta-synthesis of fathers' experiences of their partner's labour and the birth of their baby. Midwifery.

[11] NCT: MS11/AS/MN,NCTBriefing:InvolvingFathersinMaternityCare. In*.*; 2009.

[12] Miles MS, Carlson J, Funk SG (1996). Sources of support reported by mothers and fathers of infants hospitalized in a neonatal intensive care unit. Neonatal network : NN.

[13] Locock L, Alexander J: 'Just a bystander'? Men's place in the process of fetal screening and diagnosis. *Social science & medicine (1982)* 2006, 62(6):1349-1359. 10.1016/j.socscimed.2005.08.011 16165260

[14] Hilfiker-Kleiner D, Haghikia A, Masuko D, Nonhoff J, Held D, Libhaber E, Petrie MC, Walker NL, Podewski E, Berliner D *et al*: Outcome of subsequent pregnancies in patients with a history of peripartum cardiomyopathy. *European journal of heart failure* 2017:n/a-n/a. 10.1002/ejhf.808 28345302

[15] Lindberg I, Engström Å (2013). A qualitative study of new fathers experiences of care in relation to complicated childbirth. Sexual & Reproductive Healthcare.

[16] Elmir R, Schmied V (2016). A meta-ethnographic synthesis of fathers′ experiences of complicated births that are potentially traumatic. Midwifery.

[17] Steen M, Downe S, Bamford N, Edozien L (2012). Not-patient and not-visitor: a metasynthesis fathers' encounters with pregnancy, birth and maternity care. Midwifery.

[18] Patel H, Berg M, Barasa A, Begley C, Schaufelberger M (2016). Symptoms in women with Peripartum Cardiomyopathy: A mixed method study. Midwifery.

[19] Patel H, Schaufelberger M, Begley C, Berg M (2016). Experiences of health care in women with Peripartum Cardiomyopathy in Sweden: a qualitative interview study. BMC pregnancy and childbirth.

[20] Elo S, Kyngas H (2008). The qualitative content analysis process. Journal of advanced nursing.

[21] Begley CM (1996). Triangulation of communication skills in qualitative research instruments. J Adv Nurs.

[22] Meleis AI (2010). Transitions Theory: Middle Range and Situation Specific Theories in Research and Practice.

[23] Meleis AI, Sawyer LM, Im EO, Hilfinger Messias DK, Schumacher K (2000). Experiencing transitions: an emerging middle-range theory. ANS Adv Nurs Sci.

[24] Genesoni L, Tallandini MA: Men's psychological transition to fatherhood: an analysis of the literature**,** 1989-2008. *Birth (Berkeley, Calif)* 2009, 36(4):305-318. 10.1111/j.1523-536X.2009.00358.x 20002423

[25] Johansson M, Rubertsson C, Radestad I, Hildingsson I (2013). Improvements of postnatal care are required by Swedish fathers. International journal of health care quality assurance.

[26] SFOG and SBF: Antenatal care, sexual and reproductive Health. Report No. 76 from the expert panel (In Swedish) [Mödrahälsovård, Sexuell och Reproduktiv Hälsa],. In*.* Edited by SFOG TSAoOaGaTSAoM. Stockholm, Sweden; 2016.

[27] National Board of Health and Welfare: Knowledge support for maternal health. In., 2nd edn. edn. Stockholm.; 2015.

[28] Andersson E, Small R (2017). Fathers satisfaction with two different models of antenatal care in Sweden; Findings from a quasi-experimental study. Midwifery.

[29] Gage JD, Kirk R: First-time fathers: perceptions of preparedness for fatherhood. Can. J. Nurs. Res. *=Revue canadienne de recherche en sciences infirmieres* 2002, 34(4):15-24. 12619474

[30] Kunjappy-Clifton A. And father came too… A study exploring the role of first time fathers during the birth process and to explore the meaning of the experience for these men: Part two. *MIDIRS Midwifery Dig*. 2008;18

[31] ANNA J. MACHIN: Mind the Gap: The Expectation and Reality of Involved Fatherhood. *Fathering: A journal of Theory, Research, and Practice about Men as Fathers* 2015, 13(1).

[32] StGeorge JM, Fletcher RJ (2011). Fathers Online: Learning About Fatherhood Through the Internet. The Journal of perinatal education.

[33] Johansson M, Rubertsson C, Radestad I, Hildingsson I (2012). Childbirth - an emotionally demanding experience for fathers. Sexual & reproductive healthcare : official journal of the Swedish Association of Midwives.

[34] World Health Organization: Managing complications in pregnancy and childbirth: a guide for midwives and doctors. At head of title: Integrated Management of Pregnancy and Childbirth; 2007.

[35] Schumacher KL, Meleis AI (1994). Transitions: a central concept in nursing. Image J Nurs Sch.

[36] Poh HL, Koh SSL, He HG (2014). An integrative review of fathers' experiences during pregnancy and childbirth. International Nursing Review.

[37] Entsieh AA, Hallstrom IK (2016). First-time parents' prenatal needs for early parenthood preparation-A systematic review and meta-synthesis of qualitative literature. Midwifery.

[38] Shorey S, Dennis CL, Bridge S, Chong YS, Holroyd E, He HG. First-time fathers' postnatal experiences and support needs: A descriptive qualitative study. *J Adv Nurs*. 2017; 10.1111/jan.13349 28557020

[39] Ellberg L, Hogberg U, Lindh V (2010). 'We feel like one, they see us as two': new parents' discontent with postnatal care. Midwifery.

[40] Giallo R, Dunning M, Gent A (2017). Attitudinal barriers to help-seeking and preferences for mental health support among Australian fathers. Journal of Reproductive and Infant Psychology.

[41] Darwin Z, Galdas P, Hinchliff S, Littlewood E, McMillan D, McGowan L, Gilbody S (2017). Fathers' views and experiences of their own mental health during pregnancy and the first postnatal year: a qualitative interview study of men participating in the UK Born and Bred in Yorkshire (BaBY) cohort. BMC pregnancy and childbirth.

[42] Fenwick J, Bayes S, Johansson M (2012). A qualitative investigation into the pregnancy experiences and childbirth expectations of Australian fathers-to-be. Sexual & Reproductive Healthcare.

